# Composition and Factorial Invariance for the Assessment of Body Image Dissatisfaction Questionnaire in Mexican Adolescents

**DOI:** 10.3390/children8090789

**Published:** 2021-09-09

**Authors:** Raúl Josué Nájera-Longoria, José René Blanco, Carolina Jiménez-Lira, Susana Ivonne Aguirre, Humberto Blanco, Miguel Conchas-Ramírez, Yunuen Socorro Rangel-Ledezma, Perla Jannet Jurado-García

**Affiliations:** Faculty of Physical Culture Sciences, Autonomous University of Chihuahua, Chihuahua 31000, Mexico; jnajera@uach.mx (R.J.N.-L.); jblanco@uach.mx (J.R.B.); cajimenez@uach.mx (C.J.-L.); siaguirre@uach.mx (S.I.A.); hblanco@uach.mx (H.B.); mconchas@uach.mx (M.C.-R.); yrangel@uach.mx (Y.S.R.-L.)

**Keywords:** factor analysis, scale validity, body image, student beliefs, middle school education

## Abstract

Body image is a mental representation that a person has, which could become a body dissatisfaction due to the pressure exerted by the culture, affecting several life stages specially in adolescents. The aim of this study was to analyze the psychometric properties and factorial invariance of the questionnaire to assess body image dissatisfaction. The sample consisted of 552 Mexican teenagers, 259 female, and 293 male, with a mean age of 12.91 ± 0.96 years. Confirmatory Factor Analyses show that a five-factor structure is viable and adequate. The five-factor structure (perceptual, emotional distress, behavioral, proposal to change, and fear of gaining weight) show adequate fit indices and validity, even though the obtained model does not completely correspond to that proposed by the authors of the scale, it continues to endorse the multifactorial component of body dissatisfaction. On the other hand, the factorial structure, the factor loadings, and the intercepts are considered invariant in both populations; however, there are differences between the populations for the means of the perceptual, emotional distress, and fear of gaining weight factors. This study serves as a premise for future research on the study of instruments for measuring body image in populations with different personal and cultural factors.

## 1. Introduction

The earlier studies on body image go back to 1920 and are based on social and psychological perceptions of this phenomenon. Body image refers to the mental representation that the person has of his or her own body [1], and that corresponds to a changing, experiential process that is produced throughout the life cycle. Body image is especially relevant during infancy and teenage years [2,3] as its perception could be distant from the individual’s real image [4,5].

Physical activity poses a mechanism for maintaining body weight and improving health. This is the basis on which the positive influence of physical activity on body image is built [6]. In line with this, researchers have found that both the perception of one’s weight and level of fitness have an effect on behaviors directed towards obtaining or maintaining a healthy body weight [7].

The exercise intensity seems to be an important factor for physical activity, it has been pointed out that vigorous exercises offer more benefits over body fat control [8] However, a disadvantage is that this type of exercise requires previous experience and physiological adaptations. Thus, people who have not previously engaged in vigorous exercise may be discouraged from its practice and be able to obtain its beneficial impact on body image. It has also been found that people who exercise could present certain obsessive behaviors due to the cult to esthetic aspects that do not necessarily benefit their health [9]. Despite this, most studies have found that physical activity promotes a positive body image [10].

Another factor that has been found to influence body image is the use of social media, for example, significant relationships were found between the frequency of Instagram use and body dissatisfaction, drive for thinness and low self-esteem in young people [11] and depression [12]. An element that has been found to reduce the prevalence of depression during adolescence is customizing different intensities of physical activity which benefits adolescents’ academic self-efficacy by framing the positive and supportive environment in schools [13].

It has also been proposed that body dissatisfaction can be determined by cultural factors, it is believed that occidental cultures generate more pressure over the population, this has been supported by the finding that a higher level of body dissatisfaction in Latin-American teenagers [14]. Similar dissatisfaction results found in USA subjects [15], among which the white ethnic groups are more prone to these ailments [16,17] as a result, psychological problems, such as anxiety and melancholic depression, are associated with obesity in adolescence [18].

Given the importance of the construct, it is essential to be able to assess body image with valid, reliable instruments. For this reason, the present study is directed towards providing empirical support to the factorial division [19] of the questionnaire of assessment of body image dissatisfaction (IMAGEN) proposed by Solano-Pinto and Cano-Vindel [20]; this is justified by the importance of checking the factor structure of an instrument and its psychometric equivalence in different groups [21,22].

## 2. Materials and Methods

### 2.1. Participants

Five hundred and fifty-two middle-school Mexican students, 259 female and 293 male, the sample was obtained through convenience sampling (which is non-probability sampling), trying to gain representativity from different schools of the city of Chihuahua, Mexico. The size of the sample was defined like this because the Structural Equations Model methodology requires at least two hundred participants to be a representative, sample as is mentioned Ruiz, Pardo, and San Martín [23] the estimated models with sample sizes greater than 200 offer a good assurance. Inclusion criteria: Participants who resided in the city of Chihuahua, aged between 11 and 16 years, who attended secondary school, who agreed to participate in the study, and who did not have any problem that allowed them to answer the questionnaire were considered. Exclusion criteria: Participants who did not complete the questionnaire.

### 2.2. Instrument

The instrument IMAGEN Assessment of Body Image Dissatisfaction by Solano-Pinto and Cano-Vindel [20], which is made up of 38 items that allow researchers to obtain a total body image dissatisfaction score and individual scores on the cognitive-emotional (21 items), perceptual (10 items) and behavioral components (7 items).

For the present study three adaptations were made to the version by Solano-Pinto and Cano-Vindel [20]:

Consisted of changing some of the terms used in the items of the original version in order to use vocabulary that would be more appropriate to the context of the Mexican culture.

On the original scale, items included five response options: (0) rarely or never, (1) a few times, (2) sometimes I do, sometimes I don’t, (3) many times, and (4) almost always or always; in the version that was used in the present research, the participant chooses among 11 possible responses: never (0), almost never (1–3), sometimes (4–6), almost always (7–9) and always (10). This first adaptation is justified because the participants, being students, are familiar with the scale from 0 to 10, as they are evaluated that way in the educational system of our country (Mexico).

The instrument was completed using a computer. Applying the instrument in a computerized way makes it easier for the researcher to collect and refine the data with greater speed and precision.

### 2.3. Procedure

The research protocol has been approved by Scientific Committee of the Research and Postgraduate Secretariat of the Faculty of Physical Culture Sciences of the Autonomous University of Chihuahua. In addition, this research met the guidelines of the regulations of the Mexican General Health Law on Research for Health. For informed consent, contact was made with the educational authorities who oversaw speaking with the parents through each director of the institution. Once these permits were obtained, students from middle-school in the city of Chihuahua, Chihuahua, Mexico were invited to participate in the study. The questionnaire was applied in a computerized way; before accessing the instrument, participants were presented with the informed assent. To sign, the assent students pressed the “Yes I want” button, if the “I do not want” button was pressed, the system immediately abandoned the questionnaire. It was also made clear to the students that at any time they did not want to continue filling in the questionnaire, they could abandon it. The instrument described above was then completed in a single 30-min session in their school classrooms.

### 2.4. Data Analyses

The means, standard deviations, skew, and kurtosis were calculated for each item. Those with extreme skewness or kurtosis were eliminated from the scale.

Three measurement models were then compared: Model 1 (IMAGEN—3A), a three-factor model consistent with the original distribution of the questionnaire items; Model 2 (IMAGEN—3B), corresponds to the factor structure of the previous model, eliminating the items that were poorly explained; and model 3 (IMAGEN—5), a five-factor model consistent with the results obtained by Blanco and collaborators [24] in Mexican and Spanish teenagers.

Confirmatory Factor Analyses were conducted using AMOS 21 software [25]. The variances of the error terms were specified as free parameters, on each latent variable (factor) one of the associated structural coefficients was set to one in order to make the scale equal to each of the observed variables (items), according to Thompson [26].

To assess the model fit, Chi-square statistic, the Goodness of Fit Index (GFI), the Root Mean Square Residual (SRMR), and the Root Mean Square Error of Approximation (RMSEA) were used as fit measures. The Adjusted Goodness of Fit Index, the Tucker-Lewis (TLI) and the Comparative Fit Index (CFI) were used as incremental fit measures. The Chi-square over degrees of freedom ratio and the Akaike (AIC) were used as parsimony fit measures [27,28].

Reliability was then calculated for each dimension, from the best of the models (model IMAGEN—5) using Cronbach’s Alpha [29,30] and the Omega Coefficient [31,32].

In order to obtain a test that shows the best properties for the conformation of the IMAGEN questionnaire scores in male and female teenagers, a factorial invariance analysis of the obtained measurement models for the samples of male and female participants was performed, taking as the baseline model the best one (model IMAGEN—5).

## 3. Results

Participants’ age ranged between 11 and 16 years, with a mean of 12.91 and a standard deviation of 0.96. The descriptive analyses of each of the 38 questionnaire items showed that the answers to all items reflect mean scores that range between 0.64 and 5.84, and the standard deviation has, in all cases values larger than 1.96. With the exception of items 22, 24, 30, 31, 32, 33, 34, 35, 36, 37, and 38 all the other skewness and kurtosis values are within a range of ±2.00; for this reason, we infer that the variables follow a normal distribution.

### 3.1. Confirmatory Factor Analyses (CFA)

Results (GFI 0.776; RMSEA 0.074; CFI 0.880) for the model IMAGEN-3A show that is not acceptable (Table 1).

Together, the three factors from the model IMAGEN-3A explain approximately 61% of the variance. On the other hand, 9 of the 38 items possess saturations below 0.70 in their planned dimensions (items 1, 2, 3, 4, 5, 7, 10, 18 and 25). Moderate intercorrelations were observed between the three factors providing evidence of adequate discriminant validity among them.

The results from the CFA (GFI 0.900; RMSEA 0.055; CFI 0.951) for the second model (IMAGEN-3B) that correspond to the three-dimensional structure of the previous model without the items (2, 3, 5, 9, 14, 15, 20, 24, 30, 31, 36, and 37) that were not sufficiently well explained by the model IMAGEN-3A or that according to the modification indices resulted inadequately, show that this measurement model fit is barely acceptable (Table 1). Together, the factors of this model explain approximately 63% of the variance. In addition, 6 of the 26 items show saturations below 0.70 in its planned dimension (items 1, 4, 7, 18, 25, and 35). Again, moderate intercorrelations between the factors were observed, providing evidence of adequate discriminant validity among them.

The results from CFA (GFI 0.950; RMSEA 0.047; CFI 0.977) of the third and last model assessed (IMAGEN-5) that corresponds to a penta dimensional structure according to the results obtained by Blanco, Solano-Pinto, Benavides and Ornelas [24], in a sample of Mexican and Spanish teenagers, show that this measurement model fit is optimal (Table 1). Together the five factors of this model explain more than 76% of the variance. On the other hand, according to the results in Table 2, all the items show saturation above 0.70 in their planned dimension, in addition, moderate intercorrelations between the five factors were observed among the five factors providing evidence of an adequate discriminant validity among them.

### 3.2. Reliability

All resulting factors in the confirmatory factor analysis for the best model obtained (IMAGE-5) show internal consistency values over 0.75 providing evidence of a very adequate internal consistency (Table 3).

### 3.3. Confirmatory Factor Analyses for Both Samples

The results for the 19 items grouped into five factors (IMAGEN-5) in the sample of female is acceptable (GFI = 0.914; RMSEA = 0.052) and according to the incremental fit and parsimony measures which are significantly superior to the independent model and quite similar to the saturated model (Table 3). On the other hand, the confirmatory factor analyses for male indicates that the measurement model of five factors is acceptable (GFI = 0.907; RMSEA = 0.061) and according to the incremental and parsimony measures which are superior to the independent model and quite similar to the saturated model (Table 4).

According to the results shown in Table 5, in both samples, most of the items saturate over 0.70 on its planned dimension, which provides evidence of an adequate convergent validity. Moderate intercorrelations between the factors were observed providing evidence of an adequate discriminant validity among them.

### 3.4. Factorial Structure Invariance between Both Samples

The obtained fit indices (Table 6) allow for the acceptance of the equivalence of the basic measurement models between the two samples. Even though the Chi-square exceeds the required to accept the invariance hypothesis, the indices GFI = 0.911, CFI = 0.967, RMSEA = 0.40 and AIC = 727.426 contradict this conclusion which allows us to accept the baseline invariance model (no restrictions model).

Adding restrictions to the factor loadings for the baseline model we characterize the metric invariance. The values shown in Table 6 allow us to accept this level of invariance. The general fit indices (GFI 0.908) and the root mean square error of approximation (RMSEA 0.039) continue to provide convergent information in this direction. In addition, the Akaike information criteria (AIC 711.611) and the Bentler comparative index (CFI 0.967) do not show great variations with respect to the previous model.

Using the criteria for assessing nested model proposed by Cheung and Rensvold [33], who suggest that if the difference between the CFI of both nested models diminishes in 0.01 or less, the restricted model is accepted and so is factorial invariance; the difference between the obtained CFIs allows us to accept the metric invariance model. We can conclude that factor loadings are equivalent in both samples.

Once metric invariance between the samples is demonstrated, we assess intercept equivalence (strong factorial invariance). The indices (Table 6) show an optimal fit to this model both assessed independently and analyzed with respect to its nesting with the metric invariance model. The difference between the Bentler comparative indices is 0.002; the general fit index is 0.904 and the Root mean square error of approximation is 0.039. Once the strong invariance is accepted, the two assessed models are equivalents with respect to the factor coefficients and the intercepts.

All the obtained factors from the confirmatory factor analyses reached internal consistency values over 0.75 in both samples (male and female), providing evidence of an adequate internal consistency for this type of subscales, particularly when considering the reduced number of items, (Table 7).

### 3.5. Contrasts of the Factor Mean between Male and Female

Once the factorial invariance was proved, the differences between the factor mean for both groups were estimated taking as the referent the group of female participants, setting to 0 the value of the mean of that sample and freely estimating the value of the means for the sample of male teenagers. Restrictions on the regression coefficients and intercepts, which were required for the contrasts between means were automatically imposed by the AMOS 21 software [25]. Comparison results showed that the means for the perceptual, emotional distress and fear of gaining weight are significantly higher in female (−0.481. *p* < 0.05; −0.870. *p* < 0.001 and 1.006. *p* < 0.001 respectively). On the behavioral and planning of change factors, no significant differences were found.

## 4. Discussion

The goal of the present study was to obtain data about the factor structure of the questionnaire to assess body image dissatisfaction (IMAGEN) proposed by Solano-Pinto and Cano-Vindel [20] in a sample of female and male Mexican teenagers. The analyses showed that the model IMAGEN-5 has a penta factorial structure: (a) Perceptual, with 6 items; (b) Emotional distress, with 5 items; (c) Behavioral, with 2 items; (d) Planning of change, with 3 items; and (e) Fear of gaining weight, with 3 items, and is a valid and viable instrument to be used with male and female teenagers. Results that are consistent with those obtained by Blanco, Solano-Pinto, Benavides and Ornelas [24] in a similar sample of teenagers.

However, the obtained model differs to some degree with the one proposed by Solano-Pinto and Cano-Vindel [20], in some prior studies [34] although the authors report good properties of the instrument, it is observed that there is a series of items with factor loadings that are not sufficiently adequate, this could indicate that there are more factors for the questionnaire. However, in the present research, given that it shows a better fit and a greater discrimination capacity we removed half of the 38 analyzed items and change the original saturation of some of them. This action was done based on the modification indices from the confirmatory factor analyses and it is theoretically justified. However, it is important to note that the perceptual and behavioral components were kept, although with a smaller number of items, while the cognitive-emotional component was broken down into three: Emotional distress, fear of gaining weight and planning of change (named as such in this research due to the content of the items that corresponded to each component. In this way, allusion is made to the perceptual, cognitive, emotional, and behavioral components described by various authors that support a multifactorial model [35,36,37,38].

In addition, results from the factorial invariance analyses between male and female samples show high consistency between pairs of factors. This points to the existence of strong evidence of cross-validation of the measure and therefore of the structure stability until the contrary is proved. Furthermore, group comparisons reflect significant differences in three of the five assessed factors (perceptual, emotional distress, fear of gaining weight), which seems to indicate that teenage girls, in comparison to their male counterparts tend to present higher levels of dissatisfaction with their body image. This is consistent with prior research which shows that female, compared to same-aged male who report similar levels of education, frequently wish to be slimmer or lose weight independently of whether it is necessary or not [39,40,41]; and they show less satisfaction with their body image [42,43,44].

However, the scope of these results is limited, and it is necessary that future research confirms the obtained structure, which will provide more robust evidence with respect to the factor structure of the questionnaire. More studies are necessary in order to corroborate or refute the data obtained in the research completed up to this moment.

It is also essential to ascertain whether the questionnaire is useful, for example, in predicting low self-esteem, risk of eating disorders and adherence to starting and maintaining an active behavior. Since the development of body image constitutes one of the most important psychological experiences for the human being, associated with the quality of life [45] and human health [46], there is a need to valid and reliable instruments for its assessment. For this reason, the present study analyzed the psychometric properties proposed by Solano-Pinto and Cano-Vindel [20] for the IMAGEN questionnaire. In addition, this study serves as a premise for future research on the study of instruments for measuring body image in populations with different personal and cultural factors. Finally, this instrument can be widely used for application in different areas of research such as, descriptive or intervention studies.

## Figures and Tables

**Table 1:** Fit indices for models IMAGEN-3A, IMAGEN-3B, and IMAGEN-5.

	Absolute Indices				Incremental Indices			Parsimony Indices	
Model	χ^2^	GFI	RMSEA	SRMR	AGFI	TLI	CFI	CMIN/DF	AIC
IMAGEN-3A	2643.542 *	0.776	0.074	0.052	0.750	0.872	0.880	3.993	2801.542
IMAGEN-3B	784.869 *	0.900	0.055	0.036	0.880	0.946	0.951	2.670	898.869
IMAGEN-5	308.199	0.950	0.047	0.025	0.924	0.972	0.977	2.201	408.199

Note: * *p* < 0.05. GFI = Goodness of Fit Index; RMSEA = Root Mean Square Error of Approximation; SRMR = Standardized Root Mean Square Residual; AGFI = Adjusted Goodness of Fit Index; TLI = Tucker-Lewis index; CFI = Comparative Fit Index; CMIN/DF = Chi Square over Degrees of Freedom Ratio; AIC = Akaike Information Criteria.

**Table 2:** Factorial weights and correlations for the model IMAGEN-5.

Item	F1	F2	F3	F4	F5
Factorials Weights					
26	0.78				
27	0.83				
28	0.84				
29	0.91				
30	0.75				
31	0.82				
8		0.84			
9		0.87			
10		0.73			
11		0.77			
19		0.81			
37			0.90		
38			0.82		
1				0.88	
2				0.72	
5				0.71	
13					0.79
14					0.82
15					0.86
Factor Correlations					
F1					
F2	0.76				
F3	0.63	0.45			
F4	0.54	0.67	0.34		
F5	0.63	0.80	0.38	0.70	

Note: F1 = Perceptual, F2 = Emotional distress, F3 = Behavioral, F4 = Planning of change, F5 = Fear of gaining weight.

**Table 3:** Coefficient’s omega and alpha for the factors for model IMAGEN-5.

Factor	Ω	α
Perceptual	0.926	0.927
Emotional distress	0.902	0.898
Behavioral	0.851	0.845
Planning of change	0.816	0.837
Fear of gaining weight	0.864	0.860

Note: Adequate internal consistency > 0.75; Ω = Omega coefficient, and α = Cronbach’s Alpha.

**Table 4:** Fit indices CFA for both samples.

	Absolute Indices			Incremental Indices			Parsimony Indices	
Model	χ^2^	GFI	RMSEA	AGFI	TLI	CFI	CMIN/DF	AIC
Factorial solution for female								
IMAGEN-5	236.004 *	0.914	0.052	0.884	0.966	0.972	1.686	336.004
Saturated	0.000	1.000				1.000		380.000
Independent	3626.981 *	0.189	0.280	0.099	0.000	0.000	21.210	3664.981
Factorial solution for male								
IMAGEN-5	291.422	0.907	0.061	0.874	0.954	0.962	2.082	391.422
Saturated	0.000	1.000				1.000		380.000
Independent	4206.036	0.191	0.284	0.101	0.000	0.000	24.597	4244.036

Note: * *p* < 0.05. GFI = Goodness of Fit Index; RMSEA = Root Mean Square Error of Approximation; AGFI = Adjusted Goodness of Fit Index; TLI = Tucker-Lewis index; CFI = Comparative Fit Index; CMIN/DF = Chi Square over Degrees of Freedom Ratio; AIC = Akaike Information Criteria.

**Table 5:** Factorial weights and correlations for the model IMAGEN-5. Both samples.

	Female					Male				
Item	F1	F2	F3	F4	F5	F1	F2	F3	F4	F5
Factorial weights										
26	0.72					0.85				
27	0.82					0.85				
28	0.80					0.89				
29	0.88					0.94				
30	0.74					0.76				
31	0.78					0.86				
8		0.87					0.79			
9		0.90					0.82			
10		0.71					0.74			
11		0.77					0.76			
19		0.80					0.81			
37			0.90					0.89		
38			0.84					0.81		
1				0.88					0.86	
2				0.77					0.68	
5				0.76					0.66	
13					0.87					0.70
14					0.81					0.81
15					0.89					0.83
Factor Correlations										
F1										
F2	0.78					0.73				
F3	0.58	0.39				0.69	0.55			
F4	0.58	0.70	0.24			0.50	0.67	0.45		
F5	0.64	0.79	0.37	0.71		0.62	0.81	0.42	0.70	

Note: F1 = Perceptual, F2 = Emotional distress, F3 = Behavioral, F4 = Planning of change, F5 = Fear of gaining weight.

**Table 6:** Fit indices for each of the models tested for factorial invariance.

Model	Fit Index						
	χ^2^	df	GFI	NFI	CFI	RMSEA	AIC
Unrestricted Model	527.426 *	280	0.911	0.933	0.967	0.040	727.426
Metric Invariance	539.611 *	294	0.908	0.931	0.967	0.039	711.611
Strong Factorial Invariance	570.520 *	309	0.904	0.927	0.965	0.039	712.520

Note: * *p* < 0.05. GFI = Goodness of Fit Index; RMSEA = Root Mean Square Error of Approximation; AGFI = Adjusted Goodness of Fit Index; TLI = Tucker-Lewis index; CFI = Comparative Fit Index; CMIN/DF = Chi Square over Degrees of Freedom Ratio; AIC = Akaike Information Criteria.

**Table 7:** Coefficient’s omega and alpha for both samples.

	Female		Male	
Factor	Ω	α	Ω	α
Perceptual	0.909	0.909	0.944	0.945
Emotional distress	0.906	0.902	0.889	0.888
Behavioral	0.862	0.856	0.840	0.835
Planning of change	0.846	0.860	0.780	0.814
Fear of gaining weight	0.893	0.890	0.825	0.818

Note: Adequate internal consistency > 0.75; Ω = Omega coefficient, and α = Cronbach´s Alpha.

## Data Availability

Data available upon request from correspondence author.
